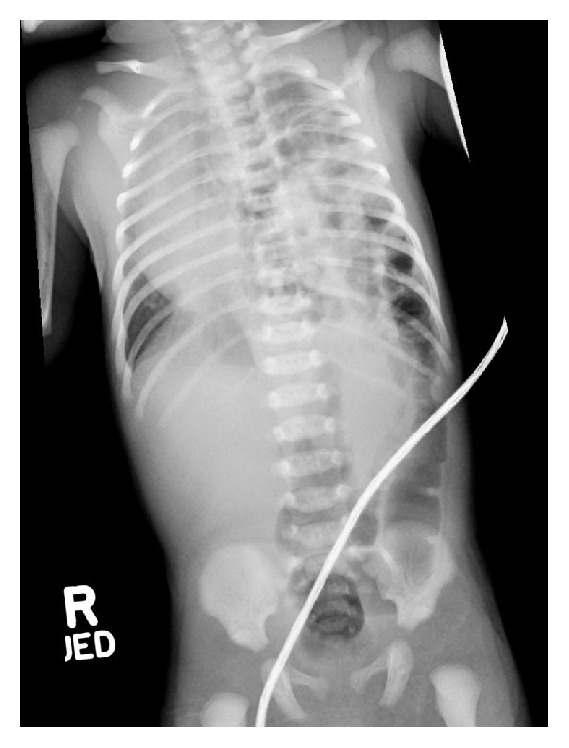# Congenital Diaphragmatic Hernia Presenting in a 7-Day-Old Infant

**DOI:** 10.1155/2017/9175710

**Published:** 2017-01-04

**Authors:** Christopher Rouse, Luke Schmidt, Lee Brock, Angela Fagiana

**Affiliations:** ^1^Department of Pediatrics, Walter Reed National Military Medical Center, Bethesda, MD, USA; ^2^Department of Pediatrics, Uniformed Services University of Health Sciences, Bethesda, MD, USA; ^3^779th Medical Operations Squadron, Joint Base Andrews, Prince George's County, MD, USA; ^4^Drexel University College of Medicine, Philadelphia, PA, USA; ^5^United States Air Force Medical Service, Washington, DC, USA

## Abstract

A 7-day-old male infant presented to the emergency room after respiratory distress was noted at an outpatient well child check. On exam, he was observed to have tachypnea, increased work of breathing, and decreased breath sounds on the left side of the chest. On chest X-ray, he was found to have a left-sided congenital diaphragmatic hernia. The infant was transported to a tertiary care facility where the defect was repaired without complication. Interestingly, the mother had a history of a normal antenatal ultrasound, completed at 19 + 2 weeks of gestational age. This case report summarizes the challenges of diagnosing late-presenting congenital diaphragmatic hernia, associated malformations, possible etiologies, and prognosis.

## 1. Background

Congenital diaphragmatic hernia (CDH) is a rare condition with an incidence of 1 in 2,000 to 1 in 4,000 live births [1]. Up to 80% to 95% of cases are diagnosed by antenatal ultrasound with many of the remaining cases diagnosed within the first 24 hours of life [2, 3]. Rarely, patients develop herniation through a diaphragmatic defect beyond the immediate newborn period. In these cases, the diagnosis of CDH may be unsuspected, particularly if the patient has a history of a normal fetal ultrasound and newborn course. This case illustrates one such example and reminds providers to stay vigilant for the diagnosis of late-presenting CDH.

## 2. Case Presentation

A 7-day-old male infant presented to the emergency room after respiratory distress was noted at an outpatient well child check. On history, the parent reported increased work of breathing starting the previous day. On exam, the infant was in respiratory distress with a respiratory rate of 70 breaths per minute, heart rate of 161 beats per minute, blood pressure of 92/47, and had a pulse oximetry reading of 91% on room air. He was noted to have subcostal and intercostal retractions, cyanotic extremities, and decreased breath sounds on the left side of the chest. His past medical history was significant for caesarian delivery at 39 + 3 weeks for failure to progress. His APGARs were 7 (1 minute) and 8 (5 minutes) and he had a birth weight of 3070 g. At birth, he was asymptomatic but was admitted briefly to the neonatal intensive care unit for a sepsis evaluation due to suspected maternal chorioamnionitis. He received 48 hours of antibiotics and was discharged home on day-of-life three. On day-of-life five, he was seen for postdischarge follow-up and had a normal exam. Of note, the mother had an antenatal ultrasound completed at 19 + 2 weeks that demonstrated normal fetal anatomy.

In the emergency room, an arterial blood gas was drawn with pH = 7.31, pCO_2_ = 53, pO_2_ = 37, and base excess = +1. CBC showed white blood cell count = 12.4K, hematocrit = 39.1%, platelets = 230K, and a differential of 50 segmented cells, 33 lymphocytes, and 11 monocytes. A chest X-ray was obtained and demonstrated a left-sided congenital diaphragmatic hernia (Figure 1).

He was transported on nasal cannula to a tertiary care facility for further treatment. Upon surgical exploration at the tertiary care facility, a 20 mm by 15 mm defect in the posterolateral diaphragm was discovered. Herniated transverse colon and small bowel were reduced and the defect was primarily closed. The infant had a benign hospital course and on day-of-life 13, he was discharged home. After surgery, the infant had no further respiratory or gastrointestinal complications.

## 3. Discussion

Because of the rarity of initial presentation in the outpatient setting, late-presenting CDH can be a challenging diagnosis. It is oftentimes not suspected which can lead to misinterpretation of radiographic imaging. Previous case reports and series have described late-presenting CDH as being misdiagnosed as a pleural effusion, pneumonia, pneumothorax, pneumatocele, and abscess [2, 4]. Maintaining a high index of suspicion can be particularly difficult if a patient, like the one described, has a history of a normal antenatal ultrasound. Additional confounding factors can include a history of a normal chest radiograph or a herniation limited to only solid viscera [5]. In one review of 122 articles and 349 children, 25% of patients with late-presenting CDH had misinterpreted radiographs [2]. In another study, 3 of the 15 patients presenting after 2 months of life were treated inappropriately with chest drains [4].

In addition to confusing imaging, patients with late-presenting congenital diaphragmatic hernia can present with a wide variety of symptoms. In acute cases, children can present with pulmonary compression and respiratory distress (such as in our patient) or with severe symptoms of bowel strangulation or volvulus [5]. Conversely, children with late-presenting CDH can also present with chronic symptoms. In one case series, 30% of patients presented with protracted gastrointestinal and respiratory symptoms such as recurrent respiratory infections, wheezing, fussiness, poor growth, intermittent abdominal pain, vomiting, progressive dyspnea, and retrosternal chest pain [4].

While our patient did not have any coexisting malformations, it is also important to have a high index of suspicion for other abnormalities in any patient who presents with CDH [6]. In one review of 104 patients diagnosed either antenatally or within the first 24 hours of life, 18 infants were found to have additional defects. The most common defect was congenital heart disease, followed by renal anomalies, spina bifida, Pierre-Robin syndrome, Fryns syndrome, esophageal atresia, omphalocele, and choledochal cyst [3]. In another case series of late-presenting CDH, 40% were diagnosed with mid-gut malrotation, 7% with multicystic kidney disease, and 7% with a spinal cord deformity [4].

It is interesting to note that there are differing theories for the etiology of late-presenting congenital diaphragmatic hernia. Some investigators suggest that the spleen or liver may initially occlude a diaphragmatic defect and when the spleen or liver then shifts sometime after birth, bowel is allowed to herniate and the patient becomes symptomatic. Another theory suggests that a transient increase in intra-abdominal pressure can cause viscera to herniate through a relatively small defect resulting in symptoms. In patients with intermittent or chronic symptoms, there is also debate as to whether herniation may be intermittent instead of fixed [2].

Finally, like our patient, those with late-presenting congenital diaphragmatic hernia have a better prognosis than those diagnosed antenatally. In one case series, infants diagnosed by antenatal ultrasound had a higher risk of pulmonary hypertension and increased mortality when compared to those diagnosed after birth [3]. Moreover, our patient's relatively small defect carried a much better prognosis than large defects, particularly those large enough to necessitate repair with a Gore-Tex patch [3].

## Figures and Tables

**Figure 1:**